# The Financial Impact of Inaccurate Blood Glucose Monitoring Systems

**DOI:** 10.1177/1932296817731423

**Published:** 2017-09-26

**Authors:** Kurt Fortwaengler, Enrique Campos-Náñez, Christopher G. Parkin, Marc D. Breton

**Affiliations:** 1 Roche Diabetes Care GmbH, Mannheim, Germany; 2 Center for Diabetes Technology, University of Virginia, Charlottesville, VA, USA; 3 CGParkin Communications, Inc, Boulder City, NV, USA

**Keywords:** diabetes, HbA1c, in silico, accuracy, inaccuracy, BGM, SMBG, cost, hypoglycemia, insulin, clinical impact, economic impact

## Abstract

**Objective:**

An in silico study of type 1 diabetes (T1DM) patients utilized the UVA-PADOVA Type 1 Diabetes Simulator to assess the effect of patient blood glucose monitoring (BGM) system accuracy on clinical outcomes. We applied these findings to assess the financial impact of BGM system inaccuracy.

**Methods:**

The study included 43 BGM systems previously assessed for accuracy according to ISO 15197:2003 and ISO 15197:2013 criteria. Glycemic responses for the 100 in silico adult T1DM subjects were generated, using each meter. Changes in estimated HbA1c, severe hypoglycemic events, and health care resource utilization were computed for each simulation. The HbA1c Translator modeling approach was used to calculate the financial impact of these changes.

**Results:**

The average cost of inaccuracy associated with the entire group of BGM systems was £155 per patient year (PPY). The average additional cost of BGM systems not meeting the ISO 15197:2003 standard was an estimated £178 PPY more than an average system that fulfills the standard and an estimated £235 PPY more than an average system that appears to meet the ISO 15197:2013 standard.

**Conclusion:**

There is a clear relationship between BGM system accuracy and cost, with the highest costs being associated with BGM systems not meeting the ISO 15197:2003 standard. Lower costs are associated with systems meeting the ISO 15197:2013 system accuracy criteria. Using BGM systems that meet the system accuracy criteria of the ISO 15197:2013 standard can help reduce the clinical and financial consequences associated with inaccuracy of BGM devices.

Patient blood glucose monitoring (BGM) is considered a key component of diabetes management.^1,2^ When utilized within a structured testing regimen, self-monitoring of blood glucose (SMBG) provides information about patients’ current glycemic status and the ability to obtain immediate feedback regarding the impact of behavioral and pharmacological interventions on glucose levels. However, because both clinicians and patients utilize SMBG data to make critical decisions regarding diabetes medication adjustments, it is crucial that glucose results are accurate.

Inaccurate glucose information can result in severe and even deadly consequences. Whereas erroneously low blood glucose results can lead to extended hyperglycemia, resulting in elevated HbA1c levels and long-term adverse outcomes, erroneously high blood glucose results can lead to severe hypoglycemia, either by failing to detect hypoglycemia or by prompting the user to over-correct with insulin.^3^

Use of inaccurate BGM systems has both clinical and economic consequences. Data from the T1D Exchange registry show that 11.8% of individuals reported having severe hypoglycemia resulting in loss of consciousness or seizure in the past year; severe hypoglycemia occurred in almost 1 in 5 older individuals (≥60 years) with long-standing (≥20 years) diabetes.^4^ A more recent report found a self-reported rate of severe hypoglycemia (defined as hypoglycemia resulting in seizure, loss of consciousness, or requiring third-party assistance due to weakness or confusion) of 31% in individuals with type 1 diabetes mellitus (T1DM) in the prior year.^5^

It is well recognized that the risk of severe or fatal hypoglycemia notably increases with age in elderly individuals with diabetes who are treated with insulinotropic medications.^6-8^ It has also been demonstrated that frequent episodes of hypoglycemia lead to hypoglycemia unawareness, worsening with age and duration of diabetes;^9^ individuals with hypoglycemia unawareness have a 6-fold increased risk for severe hypoglycemia.^10,11^ Moreover, frequent severe hypoglycemic events (SHE) can lead to fear, which can become a key obstacle to intensifying therapy and/or adhering to prescribed insulin regimens,^12,13^ resulting in poor metabolic control and subsequent health outcomes.^14^

The financial consequences of severe hypoglycemia are equally significant. The cost per episode is dependent on country and population; however, it is even more important to understand the definition of an “episode.” When considering only very severe events (requiring ambulance, emergency room or hospitalization), the cost may reach ₤2500 (together with a very low incidence) for some countries and populations. For mild cases, much higher incidences are observed but at a much lower cost of below ₤100 (mainly the Glucagon set).^15^ For an overall population, a typical definition of SHE is the DCCT definition (all cases requiring assistance). Here, the cost of the mild, severe, and very severe cases have to be averaged, leading to cost per case in the three-digit range.^15^

In this report, the DCCT definition of SHE is used with the average cost of ₤288 per case.^16^ An earlier study has shown the cost per case for T1DM patients assisted in the domestic setting (₤33 in 2017), by a community HCP (₤305) and by a hospital HCP (₤1045), with an estimated average cost of ₤181 per case (adjusted for inflation).^15^ In the T1DM population, the reported cost per case is much higher for Germany and Spain as compared with the UK. Investigators also reported the cost per SHE for T2DM patients, which showed much less variation between the three countries. A recent meta-analysis came to very similar results.^17^

The ISO 15197:2013 criteria for system accuracy of BGM devices provide an international standard that defines minimum performance requirements for BGM systems used by individuals with diabetes. According to the standard, ≥95% of all blood glucose results must be within ±15 mg/dL of the laboratory reference when blood glucose levels are below 100 mg/dL and within ±15% of the laboratory reference when levels are ≥100 mg/dL (so-called 15/15 accuracy).^18^ The current standard represents a “tightening” of requirements as compared with the previous 2003 ISO requirements, which specified that ≥95% of all blood glucose results must be within ±15 mg/dL of the laboratory reference when blood glucose levels are <75 mg/dL and within ±20% of the laboratory reference when levels are ≥75 mg/dL (so-called 15/20 accuracy).^19^

It is commonly assumed that BGM systems provide accurate test results when proper testing procedures are followed; however, this is not always the case. Although many regulatory agencies require that manufactures meet the current ISO 15197 performance standard as a requisite for clearance, an alarming number of currently marketed BGM systems do not meet the standard.^20^

Although the clinical impact of the inaccurate BGM systems has yet to be demonstrated in human trials, a recent in silico study of T1DM patients addressed this question in a study that utilized the UVA-PADOVA Type 1 Diabetes Simulator to assess the effect of BGM system accuracy on clinical outcomes.^21^ In this analysis, we apply findings from the study to assess the financial impact of inaccuracy of BGM devices on changes in HbA1c, annual SHE, and health care resource utilization (insulin and test strip consumption), using the HbA1c Translator model.^22^

## Methods

### HbA1c Translator Modeling Approach

The HbA1c Translator modeling approach is designed to support the development of simple predictive models that estimate the health and economic impact of changes in HbA1c values on insulin-treated diabetes patients.^22^ The tool contains 16 cost datasets and draws on medical evidence based on studies of over 1.08 million patients or 9.6 million patient years. The approach can be used with mixed populations of both T1DM and insulin-treated T2DM patients, regardless of age, gender and other demographic parameters. However, it is not appropriate for use in very special populations (eg, pediatric, gestational) or single individuals. In our analysis, we utilized findings from a recent study by Campos-Náñez and colleagues, who used the UVA/PADOVA Type 1 Diabetes Simulator to assess the effect of BGM system accuracy on clinical outcomes, including changes in HbA1c, SHE, insulin utilization, and SMBG frequency.^21^

### UVA/Padova Type 1 Diabetes Simulator

The UVA/PADOVA Type 1 Diabetes Simulator includes a population of 300 in silico subjects (100 adults, 100 adolescents, 100 children).^23^ Each virtual subject is represented by a model parameter vector, which is randomly extracted from an appropriate joint parameter distribution. The simulator has been successfully used by 32 research groups in academia and companies active in the field of T1DM.

In our recent analysis, we designed a 30-day scenario based on behavioral models, which were used to generate the glycemic response for each in silico adult T1DM subject (n = 100) in the simulator, using each glucose meter modeled in their database.^21^ The database included 43 blood glucose meters, previously assessed for accuracy by Freckmann and colleagues,^24^ and an “ideal” (error-free) meter as a reference. Thirty replicates of each subject / glucose meter combination were simulated, resulting in more than 10 000 simulated patient years. An estimated HbA1c and severe hypoglycemia events were computed for each simulation, utilizing a commonly used linear regression model relating average glucose to HbA1c^25^ and severe hypoglycemia events.^26^ Total daily insulin and daily SMBG test frequency were computed directly from the simulation output.

We identified two components of BGM system accuracy, bias and random error, which affect clinical outcomes. Whereas random error has little effect on HbA1c, it tends to increase episodes of severe hypoglycemia. However, system bias does have significant effects on all considered metrics; a positive systemic bias will reduce HbA1c but increase the number of severe hypoglycemia episodes, total daily insulin use, and number of fingersticks per day. Conversely, use of a system with a negative bias will slightly increase HbA1c but reduce hypoglycemia. In summary, we found that BGM systems compliant with current accuracy requirements (DIN EN ISO 15197:2013) have only limited impact on HbA1c, SHE, insulin utilization, and SMBG frequency, whereas systems not meeting the standards can have significant clinical influence on one or more of these outcomes.^21^

### Determination of Financial Impact

For our analysis, we utilized accuracy data from the 43 BGM systems tested by Freckmann and colleagues.^24^ Among these systems, they identified 34 that met the full ISO 15197:2003 assessment criteria. For nine out of the 43 assessed systems, a complete system accuracy assessment could not be performed because of a sensitivity to blood oxygen.^24^ For these systems, we used the existing data (180 instead of 200 measurements) and assumed the same performance for the missing data. Even with the reduced number of tests, five of the nine systems did not meet the ISO 15197:2013 assessment criteria; however, they appeared to be ISO 15197:2003 compliant. The remaining four systems also appeared to be ISO 15197:2013 compliant. All of the systems were modeled as if they had fulfilled the criteria for the full assessment. It should be noted that Freckmann and colleagues did not perform a full ISO 15197:2013 assessment (eg, only one and not the requested three lots of strips). However, we felt that if a system is not compliant with the criteria, it is enough to find one lot missing the requirements. For the other systems, we assumed they would have fulfilled the criteria also for the missing two lots. In summary, all of the systems were modeled as if they had fulfilled the criteria for the full assessment. It is probable that one or more of the systems would not have passed the full assessment. As a result, using them for our analysis may lead to an underestimation of the additional cost.

### Grouping of Systems for Comparison

Among the 43 assessed systems, 7 did not meet the ISO 15197:2003 standard (No ISO 2003 group). Following our approach (including the systems not fully assessed), we found that 14 of the 36 remaining systems did not meet the ISO 15197:2013 system accuracy criteria (ISO 2003/No ISO 2013 group).

We also wanted to analyze the potential for further benefits from even better accuracy. For this analysis, we assessed the systems that met the ISO 15197:2013 system accuracy criteria according to a more stringent accuracy metric (SAM). SAM is based on the fulfillment rate of 10/10; within ±10 mg/dL of the laboratory reference <100 mg/dL and within ±10% of the laboratory reference ≥100 mg/dL. This metric provides a reasonable basis for a “ranking” of the most accurate systems. In contrast to the ISO 2003 and ISO 2013 fulfillment rates, we found a wide variation of results even among the most accurate systems. To accommodate this variation, we used the median of 92.2% of glucose values as our cut point, which divides the 22 systems that meet the ISO 15197:2013 system accuracy criteria into two groups of 11 systems each. To be included in the ISO 2013/SAM group, ≥92.2% of blood glucose values must be within 10/10 metric. Systems that did not meet this metric were assigned to the ISO 2013/No SAM group (see Appendix A).

### Analysis

For each set of BGM systems—No ISO 2003, ISO 2003/No ISO 2013, ISO 2013/No SAM, and ISO 2013/SAM—we conducted a subsequent analysis of the in silico data set to further quantify the relationship between the average BGM system accuracy and changes in HbA1c levels, incidence of SHE per year and health care resource utilization (insulin and strip consumption) across the in silico population.

We then utilized a model built on the HbA1c Translator approach^22^ to calculate the financial impact of these changes, utilizing our T1DM population data set in conjunction with the UK cost data for a T1DM population as reported by Roze and colleagues.^16^ Results are reported in cost (British pounds [£]) per patient year (PPY). The design and methodology utilized by the HbA1c Translator Model is described in a recent publication.^22^

In addition to providing a fair estimate of average cost induced by SMBG system inaccuracy, we also wanted to understand what a worst-case scenario could look like. For this we performed a second analysis in which we investigated the worst effect of inaccuracy within each set on HbA1c, incidence of SHE, and total additional cost.

## Results

Analysis of the clinical impact of system inaccuracy of BGM devices among the 43 BGM systems examined in the Freckmann et al evaluation^24^ showed varying levels of impact on changes in HbA1c, SHE incidence, insulin consumption and blood glucose testing frequency across the in silico population, ranging from slightly reduced to clinically significant increased risk (Table 1).

**Table 1.** Absolute and Relative Change in HbA1c and SHE Incidence/Year.

Results	HbA1c change (%)	SHE change (cases PPY)	Insulin consumption change (IU/d)	SMBG frequency change (tests/d)
Baseline	8.75	2.86	41.80	8.37
Absolute change	−0.45 to 0.47*	−0.50 to 1.70*	−0.20 to 4.51*	−0.52 to 1.30*
Relative change	−5.2% to 5.4%	−17.5% to 59.5%	−0.5% to 10.8%	−6.2% to 15.5%

The table presents the upper and lower limits of change in the four categories. Extreme outcome among all BGM systems (*) is always associated with BGM systems not compliant with ISO 15197:2003 requirements.

Compared to an “ideal” system (including meter and strips) with no anticipated system inaccuracy, the average additional cost of inaccuracy associated with the entire group of BGM systems was £155 PPY (£95 to £219, depending on population). As shown in Table 2, the additional cost of an average BGM system not meeting the system accuracy criteria of the ISO 15197:2003 standard is an estimated £306 PPY (£169 to £446), which is an estimated £178 PPY (£85 to £270) more than the additional cost associated with an average system that fulfills the ISO 15197:2003 system accuracy criteria. And it is an estimated £235 PPY (£113 to £357) more expensive than an average system that meets the ISO 15197:2013 system accuracy criteria.

**Table 2.** Clinical Implications and Costs Associated With Average BGM System Accuracy.

All	ISO 2003	ISO 2013	SAM	HbA1c change (%)	SHE increase (cases PPY)	Additional insulin consumption (IU/d)	Additional fingersticks (tests/d)	Average additional cost (£ PPY)
(n = 43)				−0.03	0.36	1.59	0.32	155
	No (n = 7)			−0.15	0.80	2.63	0.64	306
	Yes (n = 36)			−0.01	0.27	1.39	0.26	128
		No (n = 14)		−0.08	0.52	2.11	0.47	216
		Yes (n = 22)		0.04	0.12	0.93	0.12	71
			No (n = 11)	0.07	0.11	1.09	0.11	79
			Yes (n = 11)	0.00	0.13	0.77	0.13	64

The total group (n = 43) is divided into the systems falling into the No ISO 2003 group (n = 7) and all other systems (n = 36). The remaining 36 systems are then divided into systems falling into the ISO 2003/No ISO 2013 group (n = 14) and all other systems (n = 22). The remaining 22 systems are then divided into the ISO 2013/No SAM group (n = 11) and the SAM group (n = 11).

The additional cost of an average system fulfilling the ISO 15197:2003 system accuracy criteria but not the ISO 15197:2013 system accuracy criteria is an estimated £216 PPY (£123 to £311). Such a system, on the other hand, is an estimated £145 PPY (£68 to £222) more expensive than an average system meeting the ISO 15197:2013 system accuracy criteria. As we have shown, the additional cost would even be greater when compared to a system fulfilling the SAM criteria.

The individual BGM systems with the greatest impact on total cost in each category were observed in the set of systems that did not meet the ISO 15197:2003 standard. As shown in Table 3, the additional cost of £597 is £157 higher than the additional cost of the worst meter from the ISO 2003/No ISO 2013 group, £320 higher than the additional cost of the worst meter from the ISO 2013/No SAM group and £453 higher than the additional cost of the worst meter from the SAM group.

**Table 3.** Clinical Implications and Costs Associated With Worst-Case BGM System Accuracy.

All	ISO 2003	ISO 2013	SAM	Increase in HbA1c (%)	Increase in SHE (cases PPY)	Additional cost (£ PPY)
(n = 43)				0.47	1.70	597
	No (n = 7)			0.47	1.70	597
	Yes (n = 36)			0.27	1.21	440
		No (n = 14)		0.24	1.21	440
		Yes (n = 22)		0.27	0.73	278
			No (n = 11)	0.27	0.73	278
			Yes (n = 11)	0.12	0.36	145

Table 3 represents the worst effect of inaccuracy within each set on HbA1c, incidence of SHE, and total additional cost. It is important to understand that the system leading to an increase in HbA1c of 0.47% is not the same as the one increasing the SHE by 1.70 cases PPY! The system leading to the highest additional cost of £597 may or may not be one of the two other systems (here, it is the system with worst effect on the SHE). In all but one case, the worst outcome in any of the groups is related to the worse of the two subgroups (eg, the highest additional cost of £278 among all systems who appeared to be ISO 15197:2013 compliant comes from a system falling into the ISO 2013/No SAM group).

### Sensitivity Analysis

The study population in this analysis consists of patients with type 1 diabetes treated with CSII therapy. Baseline values were 8.75% HbA1c, 2.86 SHE incidents PPY, 42 IU/day, and 8.4 blood glucose tests per day. Sensitivity analysis showed that the model used for calculation is consistent within the following baseline ranges: 6-15% HbA1c, 0.4-40 SHE incidents PPY, 5-500 IU/d, 1-50 blood glucose tests per day. Although this in silico study simulated a T1DM, CSII-treated population, we can assume that, at least qualitatively, these results can be extended to both T1DM and T2DM individuals using MDI, although further research is necessary to support this claim (see Appendix B).

## Discussion

Our previous analysis demonstrated that inaccurate BGM systems can result in significant increases in HbA1c, severe hypoglycemia and health care resource utilization.^21^ We also observed that system bias can have an effect on these metrics. Whereas a systemic negative bias will slightly raise HbA1c, a positive systemic bias will reduce HbA1c but increase the number of severe hypoglycemia episodes, total daily insulin use, and number of fingersticks per day.

In the current study, we calculated the additional costs associated with system inaccuracy of BGM devices. Because all current BGM systems exhibit some degree of inaccuracy, we used the average cost of the 43 systems assessed (£155 PPY) as our baseline measure. From our calculations we observed a clear relationship between BGM system accuracy and cost, with the highest costs associated with BGM systems that did not meet the ISO 15197:2003 system accuracy criteria, whereas notably lower costs were associated with all systems that met the ISO 15197:2013 system accuracy criteria. The greatest cost reductions were seen in the 11 systems that achieved our SAM criteria.

Although calculating additional costs based solely on accuracy is fairly straightforward, understanding the impact of systemic bias on cost is more complicated. As discussed earlier, use of a system with a negative bias will slightly increase HbA1c and reduce hypoglycemia; however, a positive systemic bias will reduce HbA1c but increase the number of severe hypoglycemia episodes and health care resource utilization. Given the high costs associated with severe hypoglycemia, it may seem reasonable to conclude that systems with a negative bias are, therefore, less costly. However, this is not necessarily the case due to the significant lot-to-lot variability found in less accurate BGM systems. In their 2013 study, Brazg and colleagues assessed the accuracy of 7 BGM systems, testing three test lots for each system.^27^ The investigators observed that although three of the seven systems met ISO 15197:2003 criteria, only one system met the current ISO 15197:2013 criteria. Importantly, three of the systems assessed showed notable lot-to-lot variability in bias. In one system, the bias ranged from –5.7 mg/dL or % to 5.3 mg/dL or %. The 2012 study from Baumstark et al has shown similar results for five other systems.^28^

Inconsistent bias poses a threat to patient safety. For example, an individual who is using a BGM system that has historically shown a negative bias may ignore impending hypoglycemia if the next vial of test strips purchased has a positive bias due to product changes or other factors. Moreover, significant lot-to-lot variability may be an indicator of system unreliability due to poor manufacturing practices. In a review of the US Food and Drug Administration MAUDE (Manufacturer and User Facility Device Experience) website,^29^ a significant proportion of reports pertaining to one BGM system (noncompliant with ISO 15197:2013) described adverse events that involved the need for emergency medical assistance and/or hospitalization for severe hypoglycemia due to meter inaccuracies.^30^

We used the UVA/PADOVA Type 1 Diabetes Simulator^23^ for our analysis. The metabolic model was complemented with a behavioral model,^21^ capturing subject behavior during meals (amounts, times and relationship to other meals), bolus (timing, missing) and fingerstick use (likelihood of a fingerstick in hypoglycemia/hyperglycemia, frequency, etc). The data were collected during studies where patients were observed for weeks, and were asked to follow their normal daily lives, stopping only to collect information. Carbohydrate counting errors were excluded, in part due to lack of access to granular data. Conversely, unbiased carbohydrate counting errors will have little effect on the average outcomes.

Finally, we could not consider individual test strip prices in our model. This is because not all meters are available in the same countries, and test strip prices vary significantly between (and even within) countries. Consequently, we decided to use an average price for all the systems.

## Conclusion

Use of SMBG is a fundamental component of self-management for individuals with diabetes, particularly those treated with insulin or other insulinotropic medications. Because blood glucose data are used in clinical decision-making, it is critical that these data are consistently accurate. Although no current BGM system is error-free, implementation of the ISO 15197:2013 standard can help to reduce the clinical and financial consequences associated with inaccuracy of BGM devices.

## Supplementary Material

Supplementary material
