# Supplementary material for: The Financial Impact of Inaccurate Blood Glucose Monitoring Systems
Source: J Diabetes Sci Technol. 2017 Sep 26;12(2):318–24. doi: 10.1177/1932296817731423 (PMC5851223; doi:10.1177/1932296817731423)
Supplement: Supplementary material [file Appendices_423.pdf]

APPENDICES

Appendix A: Comparing accuracy performance

The ISO assessments are not designed to rank systems. But if we want to further assess the performance of systems out of a certain accuracy class, we need a metric to divide our systems. Fulfilment rates of the assessment criteria are the obvious choice for such a ranking. But which of the criteria should we use? In the Freckmann et al. publication, we can find all results for the 15/20 (percentage of measurements within ±15 mg/dL of the laboratory reference when blood glucose levels are <75 mg/dL and within ±20% of the laboratory reference when levels are ≥75 mg/dL), 15/15 (percentage of measurements within ±15 mg/dL of the laboratory reference when blood glucose levels are <100 mg/dL and within ±15% of the laboratory reference when levels are ≥100 mg/dL), 10/10 and 5/5 criteria.

Obviously: for the 15/20 criteria, we see only relevant differences for the systems not compliant with the DIN EN ISO 15197:2003 – here, the values are below 95%. All the other systems are between 95% and 100%. Analogous, the 15/15 criteria shows only relevant differences for the systems not compliant with the DIN EN ISO 15197:2013. Due to the much better resolution, the 10/10 criteria as well as the 5/5 criteria show differences for all systems. So, those are the candidates for our sub-grouping. The resulting groups are the same for both criteria.

The median of all 22 systems compliant with the DIN EN ISO 15197:2013 is 92.2% fulfillment rate for the 10/10 criteria and 67.3% fulfillment rate for the 5/5 criteria.

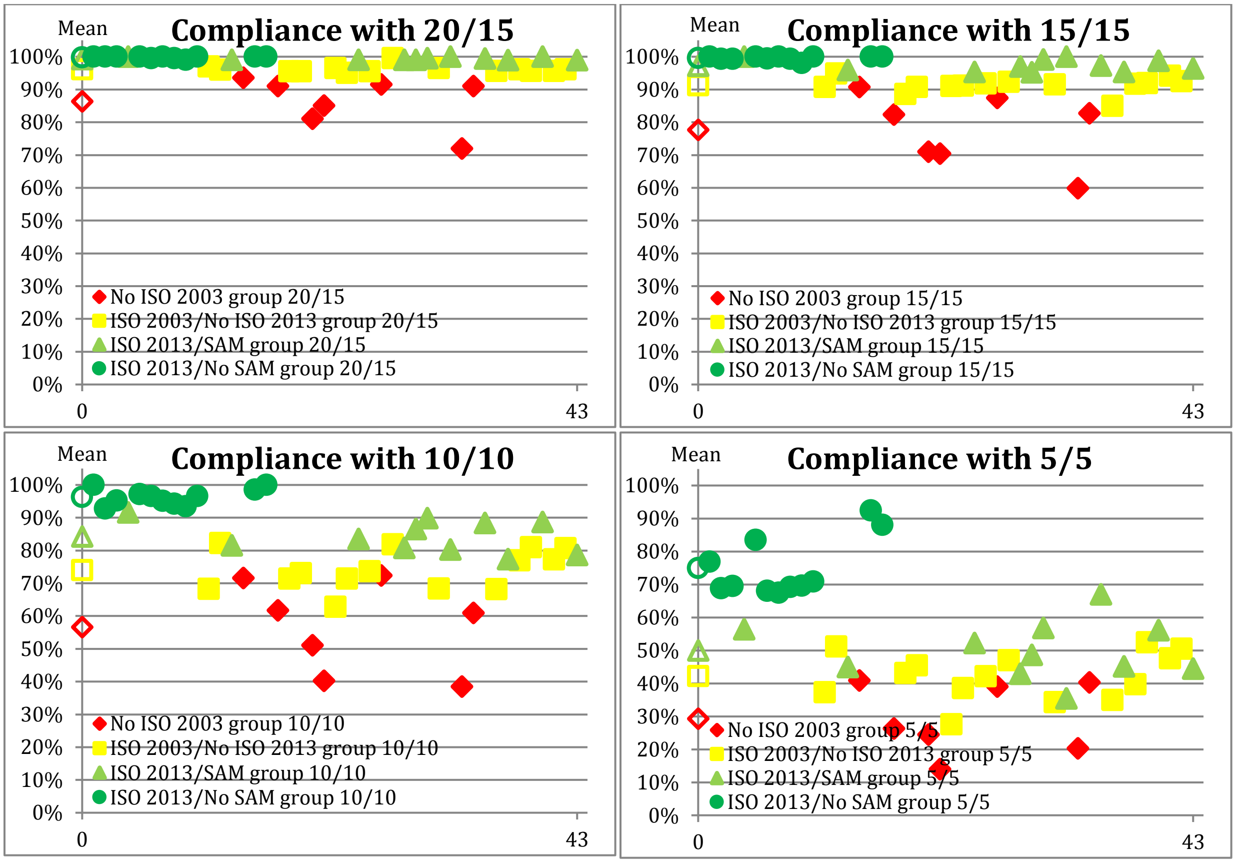

Appendix B: Model range, Sensitivity Analyses

The study population in this analysis consists of patients with type 1 diabetes treated with CSII therapy. Baseline values were: 8.75% HbA1c; 2.86 SHE incidents PPY, 42 IU/day and 8.4 blood glucose tests per day.

A. Model range

The model proposed in the previous publication is linear for HbA1c, insulin consumption and number of blood glucose tests per day but non-linear (logistic) for severe hypoglycemia. Obviously, such models have limitations: in a certain range, they will predict impossible results as negative values. We've checked the model for such limitations and found out that the results are reliable in the following ranges:

	**HbA1c** **[%]**	**SHE** [Cases PPY]	**Insulin consumption** [IU/d]	**Fingersticks** [Tests/d]
Analyzed (reliable) range	6 - 15	0.4 - 40	5 - 500	1 - 50

B. Analyzed populations

The four used variables are not independent, so a one-way sensitivity analysis as done in the previous chapter doesn't lead to realistic results. For our analysis of sensitivity and possible extremes, we used some pre-defined populations with quite extreme but not unrealistic parameters.

**Population**	**HbA1c** [%]	**SHE** [Cases PPY]	**Insulin consumption** [IU/d]	**Fingersticks** [Tests/d]	**Average Additional Cost** [£ PPY]	**Extreme Additional Cost** [£ PPY]
Baseline	8.75	2.86	41.80	8.37	155	597
Well controlled	7.50	0.50	30.00	5.00	101	362
Aggressive therapy	6.50	5.00	60.00	10.00	219	896
High HbA1c	10.00	1.50	41.80	3.00	95	294
